# Glucagon-Like Peptide-1 (GLP-1) Receptor Agonists and Thyroid Function Tests: A Systematic Review Identifying a Critical Evidence Gap

**DOI:** 10.7759/cureus.108005

**Published:** 2026-04-29

**Authors:** Sulafa Salama, Eltayeb Osman Elfaki Omer, Alaa Mahmoud, Mohamed Abdalla Mohamed ElshikhIdris, Tasneem Mohammed Mahadi Hussien, Mohammed Ahmed, Samah Osman Fadulelsayed, Sara Abdalaziz Mohammed Ibrahim, Hind Osman Ali Mohammed, Ali Hadi M Alhajri

**Affiliations:** 1 Department of Endocrinology and Diabetes, Altnagelvin Area Hospital, Londonderry, GBR; 2 Endocrinology, Dr. Sulaiman Al Habib Hospital, Riyadh, SAU; 3 Cardiology, Lincoln County Hospital, Lincoln, GBR; 4 General Medicine, Al Yarmouk College, Khartoum, SDN; 5 Internal Medicine, Pioneer Health Medical Clinics, Saham, OMN; 6 Endocrinology and Diabetes, James Cook University Hospital, Middlesbrough, GBR; 7 Endocrinology and Diabetes, NHS/Health Education England, Chelmsford, GBR; 8 Internal Medicine, Dana Medical Center, Healthline Medical Group, Abu Dhabi, ARE; 9 General Medicine, University Hospital Waterford, Waterford, IRL; 10 Endocrinology, Najran Armed Forces Hospital, Ministry of Defense Health Services, Najran, SAU

**Keywords:** free thyroxine, glp-1 receptor agonists, liraglutide, semaglutide, systematic review, thyroid function tests, thyroid safety, thyrotropin

## Abstract

Glucagon-like peptide-1 (GLP-1) receptor agonists are widely used for type 2 diabetes and obesity management, yet their effects on thyroid function tests remain inadequately characterized. Preclinical rodent studies demonstrating C-cell hyperplasia have raised safety concerns, while clinical evidence regarding thyroid hormone dynamics is sparse and inconsistent. This systematic review aimed to synthesize available evidence on the effects of GLP-1 receptor agonists on thyroid function tests, explicitly acknowledging that direct evidence is limited and that characterizing this evidence gap is a primary objective.

A comprehensive search of PubMed, Scopus, Web of Science, and ClinicalTrials.gov was conducted for studies published between January 2021 and December 2025. Eligible studies included randomized controlled trials and observational studies evaluating thyroid function parameters in adult patients receiving GLP-1 receptor agonists. Risk of bias was assessed using the Cochrane Risk of Bias 2 tool for randomized trials and the Risk Of Bias In Non-randomized Studies of Interventions tool for observational studies. A narrative synthesis was performed due to substantial methodological heterogeneity precluding meta-analysis.

Six studies encompassing 900,225 participants met the inclusion criteria, including three randomized controlled trials, two retrospective cohort studies, and one combined Mendelian randomization and cohort study. Only one study directly assessed thyroid function tests, reporting a mild decrease in free thyroxine and slight thyroid nodule progression after 12 months of therapy, though Mendelian randomization analysis revealed no significant causal effect. The remaining five studies did not systematically evaluate thyroid function parameters, focusing instead on thyroid malignancy outcomes or weight loss efficacy. The large-scale cohort studies demonstrated no increased risk of thyroid tumors or thyroid cancer with GLP-1 receptor agonist use. Risk of bias was low for two observational studies and moderate for one due to potential residual confounding, while two randomized trials demonstrated low risk of bias and one raised some concerns regarding outcome measurement.

This systematic review aims to critically evaluate the available evidence - and, more explicitly, to characterize the substantial evidence gap - concerning the effects of GLP-1 receptor agonists on thyroid function tests. While only one of six included studies directly addressed this outcome, and available data suggest no increased thyroid malignancy risk, definitive conclusions regarding functional thyroid effects cannot be drawn due to the absence of systematic thyroid function assessments in most included studies, particularly the major randomized controlled trials. Future research should incorporate systematic thyroid hormone monitoring to clarify the clinical significance of GLP-1 receptor agonist therapy on thyroid axis regulation.

## Introduction and background

Glucagon-like peptide-1 (GLP-1) receptor agonists (RAs) have emerged as a cornerstone in the management of type 2 diabetes mellitus (T2DM) and, more recently, obesity [1]. By enhancing glucose-dependent insulin secretion, suppressing glucagon release, delaying gastric emptying, and promoting satiety, these agents provide significant glycemic control alongside favorable effects on body weight and cardiovascular outcomes [2]. Owing to these benefits, the use of GLP-1 RAs has expanded rapidly in clinical practice.

Thyroid function plays a critical role in metabolic homeostasis, and even subtle alterations in thyroid hormones can significantly influence energy balance and cardiovascular health [3]. Thyroid function tests (TFTs) - including thyroid-stimulating hormone (TSH, the primary regulator of thyroid activity), free thyroxine (fT4), and triiodothyronine (T3, the active hormone) - are commonly used to assess thyroid status [4]. Biologically, GLP-1 receptors are expressed not only in pancreatic cells but also in the central nervous system, including the hypothalamus, where they may influence thyrotropin-releasing hormone (TRH) secretion. Additionally, GLP-1 receptors have been identified on thyroid C-cells (which produce calcitonin) and, to a lesser extent, on follicular cells. Thus, GLP-1 RAs could theoretically modulate the hypothalamic-pituitary-thyroid (HPT) axis through both central and peripheral mechanisms [5].

Preclinical studies have reported C-cell hyperplasia and medullary thyroid carcinoma in rodent models following GLP-1 RA exposure [6]. Although these findings have not been consistently replicated in humans, they have led to regulatory warnings and heightened surveillance of thyroid-related outcomes. Emerging clinical evidence has reported variable effects on TSH and thyroid hormone levels, with some studies indicating modest TSH reductions potentially mediated by weight loss or central neuroendocrine effects [7].

Despite growing interest, a critical knowledge gap persists: most clinical trials of GLP-1 RAs have not systematically assessed TFTs as primary or secondary outcomes. Instead, they have focused on glycemic control, weight loss, or major adverse cardiovascular events, with thyroid safety evaluated primarily through malignancy surveillance rather than functional parameters. Consequently, whether GLP-1 RAs meaningfully alter TSH, fT4, or T3 levels - and whether such changes have clinical significance - remains largely unknown. The existing evidence is heterogeneous and conflicting, with differences in study design, patient populations, and specific agents further obscuring conclusions.

Given the expanding use of GLP-1 RAs and the absence of prospective TFT assessments in most major trials, a comprehensive synthesis of available evidence is warranted. Therefore, this systematic review aims not to provide a definitive evaluation of the effects of GLP-1 RAs on TFTs, as most included studies did not assess these outcomes, but rather to explicitly characterize the evidence gap, identifying what is known and what remains unknown regarding changes in TSH, fT4, and T3 levels. By consolidating current evidence and its limitations, this review seeks to clarify the relationship between GLP-1 RA therapy and thyroid function while informing clinical practice and future research directions.

## Review

Methodology

Protocol and Reporting Guidelines

This systematic review was conducted in accordance with the Preferred Reporting Items for Systematic Reviews and Meta-Analyses (PRISMA) guidelines [8]. The methodology was carefully designed to ensure transparency, reproducibility, and methodological rigor in identifying, selecting, and synthesizing relevant studies assessing the effects of GLP-1 RAs on TFTs. A protocol was not registered in PROSPERO. While we acknowledge that protocol registration is a best practice that enhances transparency and reduces reporting bias, this review was initiated as an exploratory synthesis without a predefined hypothesis about the direction or magnitude of effects, and the anticipated scarcity of direct evidence on TFTs made specification of primary outcomes challenging. Nevertheless, the absence of prospective registration represents a limitation of this review.

Eligibility Criteria (PICOS Framework)

The eligibility criteria for study selection were defined using the Population, Intervention, Comparison, Outcomes, and Study design (PICOS) framework [9]. Only studies published within the last five years (2021-2025) were included to ensure incorporation of the most recent and clinically relevant evidence (Table 1). This specific timeframe was chosen because the majority of large-scale randomized controlled trials (RCTs) and real-world cohort studies evaluating thyroid-related outcomes with GLP-1 RAs have been published since 2021, reflecting the expanded clinical use of these agents for both diabetes and obesity indications following recent regulatory approvals.

**Table 1:** Population, Intervention, Comparison, Outcomes, and Study design (PICOS) Eligibility Criteria TSH: thyroid-stimulating hormone, GLP-1: glucagon-like peptide-1

Component	Description
Population (P)	Adult patients (≥18 years) with type 2 diabetes mellitus, obesity, or related metabolic disorders, with or without pre-existing thyroid disease
Intervention (I)	Administration of GLP-1 receptor agonists (e.g., liraglutide, semaglutide, exenatide, dulaglutide)
Comparison (C)	Placebo, standard care, or other antidiabetic/anti-obesity treatments
Outcomes (O)	Changes in thyroid function tests, including TSH, free T4 (fT4), T3, and other thyroid-related biomarkers
Study Design (S)	Randomized controlled trials (RCTs), cohort studies, and case-control studies

Studies were excluded if they were animal studies, in vitro studies, case reports, reviews, editorials, conference abstracts without full text, or studies not reporting thyroid-related outcomes.

Information Sources

A comprehensive literature search was conducted across multiple electronic databases, including PubMed, Scopus, and Web of Science. In addition, ClinicalTrials.gov was searched to identify ongoing or unpublished studies. To ensure completeness, reference lists of included studies and relevant reviews were manually screened for additional eligible articles.

Search Strategy

A structured search strategy was developed using a combination of Medical Subject Headings (MeSH) terms and free-text keywords related to GLP-1 RAs and thyroid function. The search strategy was adapted for each database (Table 2).

**Table 2:** Search Strategy

Database	Search Date	Full Search Syntax
PubMed	January 10, 2026	(("glucagon-like peptide-1 receptor agonist"[MeSH Terms] OR "GLP-1 receptor agonist"[Title/Abstract] OR "liraglutide"[Title/Abstract] OR "semaglutide"[Title/Abstract] OR "exenatide"[Title/Abstract] OR "dulaglutide"[Title/Abstract] OR "lixisenatide"[Title/Abstract])) AND (("thyroid function tests"[MeSH Terms] OR "thyroid function"[Title/Abstract] OR "TSH"[Title/Abstract] OR "thyroid stimulating hormone"[Title/Abstract] OR "thyroid hormones"[Title/Abstract] OR "T3"[Title/Abstract] OR "triiodothyronine"[Title/Abstract] OR "T4"[Title/Abstract] OR "free thyroxine"[Title/Abstract] OR "calcitonin"[Title/Abstract])) AND ((("y_5"[Filter]) AND (2021/01/01:2025/12/31[pdat])) AND (english[Filter]))
Scopus	January 10, 2026	( TITLE-ABS-KEY ( "GLP-1 receptor agonist" OR "glucagon-like peptide-1 receptor agonist" OR liraglutide OR semaglutide OR exenatide OR dulaglutide OR lixisenatide ) AND TITLE-ABS-KEY ( "thyroid function" OR "thyroid function tests" OR tsh OR "thyroid stimulating hormone" OR "thyroid hormones" OR t3 OR triiodothyronine OR t4 OR "free thyroxine" OR calcitonin ) ) AND PUBYEAR > 2020 AND PUBYEAR < 2026 AND ( LIMIT-TO ( LANGUAGE , "English" ) )
Web of Science	January 10, 2026	( TS=( "GLP-1 receptor agonist" OR "glucagon-like peptide-1 receptor agonist" OR liraglutide OR semaglutide OR exenatide OR dulaglutide OR lixisenatide ) ) AND ( TS=( "thyroid function" OR "thyroid function tests" OR tsh OR "thyroid stimulating hormone" OR "thyroid hormones" OR t3 OR triiodothyronine OR t4 OR "free thyroxine" OR calcitonin ) ) AND ( PY=(2021-2025) ) AND ( LA=(English) )
ClinicalTrials.gov	January 10, 2026	Condition: "thyroid" OR "TSH" OR "thyroid function" Intervention: "GLP-1 receptor agonist" OR "liraglutide" OR "semaglutide" OR "exenatide" OR "dulaglutide"

Filters were applied to include studies published between January 2021 and December 2025 and restricted to human subjects and English language publications.

Study Selection

All retrieved records were imported into EndNote X9 software for reference management and duplicate removal. Following deduplication, titles and abstracts were independently screened by reviewers to identify potentially eligible studies. Full texts of selected articles were then assessed for eligibility based on predefined inclusion and exclusion criteria. Any discrepancies between reviewers were resolved through discussion and consensus.

Data Extraction

Data extraction was performed using a standardized data extraction form. Two review authors independently extracted data from all included studies, and any discrepancies were resolved through discussion or consultation with a third reviewer when consensus could not be reached. Extracted information included study characteristics (author, year, country), study design, sample size, participant characteristics, type and duration of GLP-1 RA intervention, comparator details, and reported thyroid function outcomes (TSH, fT4, T3). Where necessary, corresponding authors were contacted for missing or unclear data.

Risk of Bias Assessment

The methodological quality of included studies was assessed using validated tools appropriate to study design. The Cochrane Risk of Bias 2 (RoB 2) tool [10] was used for RCTs, while the Risk Of Bias In Non-randomized Studies of Interventions (ROBINS-I) tool [11] was used for observational studies. Each study was evaluated across multiple domains, including selection bias, performance bias, detection bias, and reporting bias, to ensure a comprehensive assessment of internal validity.

Data Synthesis

A qualitative synthesis of findings was conducted to summarize the effects of GLP-1 RAs on TFTs. A meta-analysis was not performed due to substantial heterogeneity among included studies. Specifically, variations in study design, patient populations (e.g., diabetic vs. obese individuals), types and dosages of GLP-1 RAs, duration of follow-up, and differences in reported thyroid outcomes (including measurement methods and units) limited the comparability of results. Additionally, inconsistent reporting of effect sizes and insufficient availability of raw data further precluded robust quantitative pooling. Conducting a meta-analysis under such conditions could lead to misleading conclusions; therefore, a narrative synthesis was considered the most appropriate approach.

Results

Study Selection Process

**Figure 1:**
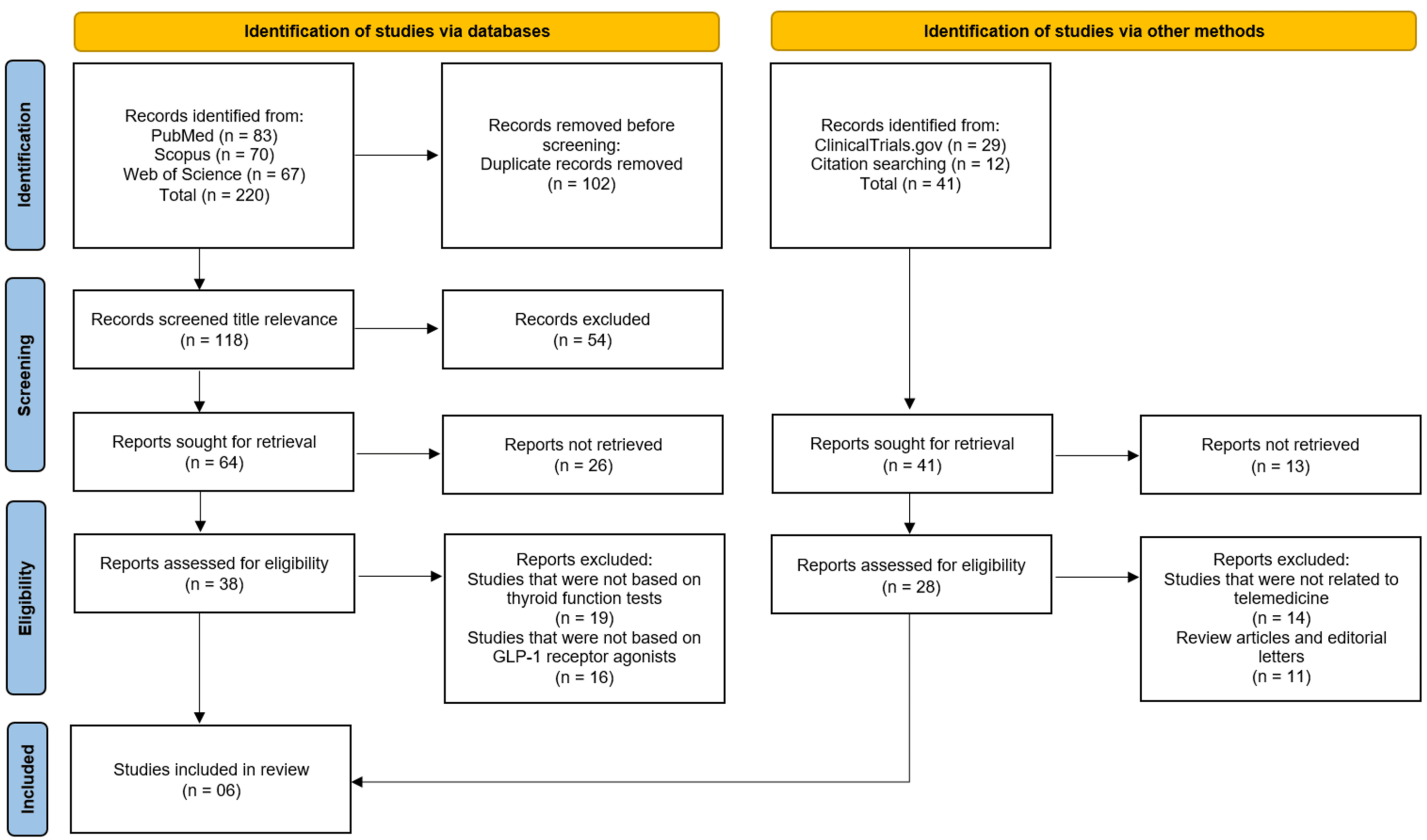
Illustration of Studies Selection on Preferred Reporting Items for Systematic Reviews and Meta-Analyses (PRISMA) Flowchart

The study selection process is illustrated in the PRISMA flow diagram (Figure 1). A comprehensive literature search was conducted across PubMed, Scopus, and Web of Science, yielding a total of 220 records, comprising 83 records from PubMed, 70 records from Scopus, and 67 records from Web of Science. Prior to screening, 102 duplicate records were identified and removed, leaving 118 records for title and abstract screening based on relevance to the research question. Following this initial screening, 54 records were excluded as they did not meet the preliminary inclusion criteria, and 64 reports were sought for retrieval. Of these, 26 reports could not be retrieved, resulting in 38 reports being assessed for full-text eligibility. We acknowledge that the inability to retrieve these 26 reports may introduce potential selection bias, as unpublished or inaccessible findings could differ systematically from those available for full-text review; however, we did contact the corresponding authors but got no response. During the eligibility assessment, 19 reports were excluded because they did not assess TFTs as an outcome measure, and an additional 16 reports were excluded because the intervention was not based on GLP-1 RAs. Concurrently, identification of studies via other methods included searching ClinicalTrials.gov, which yielded 29 records, and citation searching of relevant articles, which identified 12 additional records, totaling 41 records from alternative sources. Of these, 13 reports were not retrieved, leaving 28 reports for full-text eligibility assessment. Upon detailed review, 14 reports were excluded as they were unrelated to the core topic, and 11 reports were excluded because they were review articles or editorial letters rather than primary research. Ultimately, a total of six studies met all inclusion criteria and were included in this systematic review [12-17].

Study Selection and Characteristics

The systematic review included six studies comprising a total of 900,225 participants across various study designs, including Mendelian randomization analyses, retrospective cohort studies, and RCTs. The characteristics of all included studies are summarized in Table 3. The included studies were published between 2021 and 2026 and were conducted across multiple countries spanning Asia, Europe, and North America. Among the six included studies, three were RCTs [15-17], two were large-scale retrospective cohort studies [13,14], and one employed a combined Mendelian randomization and cohort study design [12]. The duration of follow-up varied considerably across studies, ranging from 44 weeks in the RCT by Mu and colleagues [15] to a mean follow-up period of 3.9 years in the Scandinavian registry-based cohort study by Pasternak and colleagues [14].

**Table 3:** Characteristics of Included Studies GLP-1: glucagon-like peptide-1, fT4: free thyroxine, TIRADS: Thyroid Imaging Reporting and Data System, T2DM: type 2 diabetes mellitus, ITT: intention-to-treat, DPP-4: dipeptidyl peptidase-4, SGLT2i: sodium-glucose cotransporter 2 inhibitors, SUs: sulfonylureas, GI: gastrointestinal, AEs: adverse events, RCT: randomized controlled trial, BMI: body mass index, WC: waist circumference, BP: blood pressure, HbA1c: hemoglobin A1c, CRP: C-reactive protein, QoL: quality of life, DXA: dual-energy X-ray absorptiometry, SF-36: 36-Item Short Form Health Survey

Author (Year)	Country	Study Design	Sample Size (n)	Population Characteristics	GLP-1 Receptor Agonist	Comparator	Duration of Follow-up	Outcome Measures	Key Findings
Zhang et al., [12] (2026)	China	Mendelian randomization analysis + cohort study	169	Patients with diabetes mellitus	GLP-1 receptor agonists	No GLP-1 treatment / standard antidiabetic therapy	12 months	fT4, thyroid nodule diameter, TIRADS classification	12-month use linked to mild ↓ fT4 and slight nodule progression; no significant causal effect in regression; overall safe
Morales et al., [13] (2025)	US, Germany, Spain, UK	Retrospective cohort	316,587–460,032	Adults with T2DM on metformin	Various GLP-1 receptor agonists	SGLT2i, DPP-4i, SU	ITT & on-treatment	Thyroid tumors	No increased thyroid tumor risk vs SGLT2i, DPP-4i, SUs
Pasternak et al., [14] (2024)	Denmark, Norway, Sweden	Nationwide registry-based cohort	437,077	Adults 18–84 yrs with T2DM; cancer-free at baseline	Liraglutide, semaglutide, dulaglutide, exenatide, lixisenatide	DPP-4 inhibitors	Mean 3.9 yrs	Incident thyroid cancer; HRs via Cox models	No increased thyroid cancer risk vs DPP-4 or SGLT2i; no subtype risk increase
Mu et al., [15] (2024)	China, Hong Kong, Brazil, South Korea	Randomized, double-blind, phase 3a, multicentre controlled trial	375 randomized	Adults with overweight or obesity, with or without T2DM; predominantly East Asian population	Semaglutide 2.4 mg once weekly	Placebo	44 weeks	Percentage change in body weight; proportion achieving ≥5% weight loss; safety outcomes	Significant weight loss vs placebo (−12.1% vs −3.6%, p<0.0001); thyroid markers not assessed; GI AEs most common
Wadden et al., [16] (2021)	USA	RCT, double-blind, placebo-controlled, multicenter	611 (407/204)	Adults with overweight/obesity; no diabetes; mean age 46; 81% female; mostly White; cardiometabolic comorbidity common	Semaglutide 2.4 mg weekly	Placebo	68 weeks + 7-week follow-up	Weight change, ≥5/10/15/20% loss, WC, BP, SF-36, HbA1c, glucose, lipids, CRP, AEs	No thyroid labs reported; 1 papillary thyroid cancer case; no hormone/calcitonin assessment
Wilding et al., [17] (2021)	Multinational (16 countries)	RCT, double-blind, placebo-controlled	1961 (1306 vs 655)	Adults, BMI ≥30 or ≥27 + comorbidities; non-diabetic; mean age 46; 74% female	Semaglutide 2.4 mg weekly	Placebo + lifestyle	68 + 7 weeks	Weight change, % weight loss, BMI, WC, BP, HbA1c, lipids, CRP, QoL, DXA fat/lean mass, AEs	Weight loss, metabolic benefits; no thyroid tests assessed

Study Populations and GLP-1 RA Exposure

The study populations demonstrated heterogeneity in terms of baseline characteristics and indications for GLP-1 RA use. Morales and colleagues [13] included 316,587 to 460,032 adults with T2DM on metformin across the United States, Germany, Spain, and the United Kingdom, comparing various GLP-1 RAs with sodium-glucose cotransporter-2 inhibitors, dipeptidyl peptidase-4 inhibitors, and sulfonylureas. Similarly, Pasternak and colleagues [14] conducted a nationwide registry-based cohort study across Denmark, Norway, and Sweden, encompassing 437,077 adults aged 18 to 84 years with T2DM who were cancer-free at baseline, with exposure to liraglutide, semaglutide, dulaglutide, exenatide, and lixisenatide compared against dipeptidyl peptidase-4 inhibitors. In contrast, three RCTs focused on weight management populations without diabetes or with overweight and obesity. The STEP 7 trial by Mu and colleagues [15] randomized 375 participants from China, Hong Kong, Brazil, and South Korea with predominantly East Asian ethnicity to receive semaglutide 2.4 mg once weekly or placebo. The STEP 3 trial by Wadden and colleagues [16] included 611 adults with overweight or obesity without diabetes, with a mean age of 46 years and predominantly White ethnicity with 81% female representation. The largest RCT by Wilding and colleagues [17] enrolled 1,961 participants across 16 countries, including adults with a body mass index of 30 or greater or 27 or greater with comorbidities, without diabetes. The study by Zhang and colleagues [12] included 169 patients with diabetes mellitus in their cohort analysis component, evaluating GLP-1 RA use compared with standard antidiabetic therapy.

Effects on TFTs

Assessment of TFTs was limited across the included studies, with only one study specifically designed to evaluate thyroid-related outcomes as primary endpoints. Zhang and colleagues [12] reported that 12-month use of GLP-1 RAs was associated with a mild decrease in free thyroxine levels and slight thyroid nodule progression based on Thyroid Imaging Reporting and Data System classification. Specifically, the study documented a mean reduction in free thyroxine of approximately 0.08 ng/dL (from baseline values within the normal range), representing a modest but statistically significant change that remained well within population reference intervals. No significant alterations in TSH or T3 levels were observed. However, the Mendelian randomization analysis component of this study revealed no significant causal effect in the regression analysis, suggesting that the observed associations may not reflect direct causality. Importantly, the authors concluded that GLP-1 RA use appeared overall safe with respect to thyroid function, noting that the small magnitude of free thyroxine change was unlikely to be clinically meaningful in euthyroid individuals [12]. However, this finding derives from a single study and should therefore be interpreted with caution given the limited evidence base and the lack of corroborating data from other investigations.

The remaining five studies did not systematically assess TFTs as outcome measures. In the large retrospective cohort studies by Morales and colleagues [13] and Pasternak and colleagues [14], the primary outcomes focused on incident thyroid tumors and thyroid cancer risk rather than functional thyroid parameters. Similarly, the three RCTs evaluating semaglutide for weight management did not include TFTs in their reported outcomes. Mu and colleagues [15] focused on percentage change in body weight and the proportion of participants achieving clinically meaningful weight loss thresholds, without assessment of thyroid markers. Wadden and colleagues [16] reported on weight change outcomes, cardiometabolic parameters, and safety events but explicitly stated that no thyroid laboratory assessments were performed, and no hormone or calcitonin measurements were obtained. Wilding and colleagues [17] similarly did not include TFTs among their comprehensive metabolic and body composition assessments.

Critically, the absence of TFT data in five of six studies has two major implications. First, it precludes any quantitative synthesis or meta-analysis of GLP-1 RA effects on TSH, fT4, or T3 levels, leaving the true effect size, if any, unknown. Second, it introduces substantial ascertainment bias, as studies not designed to measure TFTs may have undetected thyroid dysfunction events that went unreported. Thus, while the available evidence from Zhang and colleagues [12] suggests only minimal changes in free thyroxine with no causal evidence, the overall literature cannot rule out clinically significant thyroid effects due to systematic non-assessment of this outcome domain.

Thyroid Nodule and Cancer Outcomes

Three studies specifically evaluated the risk of thyroid nodules or thyroid malignancies associated with GLP-1 RA use. Zhang and colleagues [12] observed slight nodule progression based on Thyroid Imaging Reporting and Data System classification after 12 months of GLP-1 RA exposure, though this finding was not corroborated by causal evidence in their Mendelian randomization analysis. The study did not report thyroglobulin or calcitonin measurements, which could have provided additional insight into thyroid tumor activity or C-cell proliferation. In contrast, the large-scale retrospective cohort study by Morales and colleagues [13] found no increased risk of thyroid tumors with GLP-1 RA use compared with sodium-glucose cotransporter-2 inhibitors, dipeptidyl peptidase-4 inhibitors, or sulfonylureas in both intention-to-treat and on-treatment analyses. Similarly, Pasternak and colleagues [14] reported no increased risk of incident thyroid cancer associated with GLP-1 RA use compared with dipeptidyl peptidase-4 inhibitors or sodium-glucose cotransporter-2 inhibitors over a mean follow-up of 3.9 years, with hazard ratios derived from Cox proportional hazards models demonstrating no subtype-specific risk increases. Among the RCTs, only Wadden and colleagues [16] reported a single case of papillary thyroid cancer in their safety outcomes, though formal hormone or calcitonin assessments were not conducted.

Weight Loss and Metabolic Outcomes

The three RCTs included in this systematic review consistently demonstrated significant weight loss benefits with semaglutide 2.4 mg once weekly compared with placebo. Mu and colleagues [15] reported a mean weight change of −12.1% with semaglutide compared with −3.6% with placebo in a predominantly East Asian population, a difference that was highly statistically significant. Wilding and colleagues [17] similarly documented substantial weight loss, improvements in body mass index, waist circumference, blood pressure, glycemic parameters, lipid profiles, C-reactive protein levels, and dual-energy X-ray absorptiometry-derived fat and lean mass measurements with semaglutide treatment. Wadden and colleagues [16] corroborated these findings, reporting significant weight loss and improvements in cardiometabolic risk factors including waist circumference, blood pressure, and quality of life measures. Gastrointestinal adverse events were consistently the most commonly reported safety concern across all three trials [15-17].

Risk of Bias Assessment

The risk of bias was assessed using the ROBINS-I tool for the three observational studies [12-14] and the RoB 2 tool for the three RCTs [15-17], with the detailed judgments presented in Table 4 and Table 5, respectively. Among the non-randomized studies, the combined Mendelian randomization and cohort study by Zhang and colleagues [12] and the retrospective cohort study by Morales and colleagues [13] were both judged to have a low overall risk of bias across all domains, including confounding, selection of participants, classification of interventions, deviations from intended interventions, missing data, measurement of outcomes, and selection of the reported result. The nationwide registry-based cohort study by Pasternak and colleagues [14] was assessed as having a moderate overall risk of bias, with concerns specifically noted in the confounding domain due to the potential for residual confounding inherent to observational designs, while all other domains were judged to be at low risk of bias. For the RCTs evaluated using the RoB 2 tool, the trial by Mu and colleagues [15] and the trial by Wilding and colleagues [17] demonstrated a low overall risk of bias, with low risk ratings assigned to the randomization process, deviations from intended interventions, missing outcome data, measurement of the outcome, and selection of the reported result. In contrast, the STEP 3 trial by Wadden and colleagues [16] was judged to have some concerns overall, driven by a rating of some concerns in the measurement of the outcome domain, specifically regarding the absence of thyroid laboratory or hormonal assessments, although all other domains were rated as low risk.

**Table 4:** Risk of Bias Assessment for Non-Randomized Studies (ROBINS-I) [11]

Study	Confounding	Selection of Participants	Classification of Interventions	Deviations from Intended Interventions	Missing Data	Measurement of Outcomes	Selection of Reported Result	Overall Risk of Bias
Zhang et al., [12] (2026)	Low	Low	Low	Low	Low	Low	Low	Low
Morales et al., [13] (2025)	Low	Low	Low	Low	Low	Low	Low	Low
Pasternak et al., [14] (2024)	Moderate	Low	Low	Low	Low	Low	Low	Moderate

**Table 5:** Risk of Bias Assessment for Randomized Controlled Trials (RoB 2) [10]

Study	Randomization Process	Deviations from Intended Interventions	Missing Outcome Data	Measurement of the Outcome	Selection of the Reported Result	Overall Risk of Bias
Mu et al., [15] (2024)	Low	Low	Low	Low	Low	Low
Wadden et al., [16] (2021)	Low	Low	Low	Some concerns	Low	Some concerns
Wilding et al., [17] (2021)	Low	Low	Low	Low	Low	Low

Discussion

Summary of Principal Findings

This systematic review reveals a striking discordance between the widespread clinical use of GLP-1 RAs and the availability of robust data regarding their effects on TFTs. Only one of six included studies directly assessed TFTs as an outcome [12], while the remaining five focused on thyroid malignancy [13,14] or weight loss endpoints without thyroid laboratory assessments [15-17]. Thus, the overarching conclusion is one of evidence paucity rather than evidence certainty.

Evidence From the Single Study Assessing TFTs

The most direct evidence emanates from Zhang et al. [12], who reported a mild decrease in fT4 (approximately 0.08 ng/dL, remaining within normal range) and slight thyroid nodule progression following 12 months of GLP-1 RA exposure. However, their concurrent Mendelian randomization analysis failed to demonstrate a significant causal relationship, suggesting that observed associations may reflect residual confounding or metabolic perturbations inherent to diabetes rather than a direct pharmacological effect. Importantly, the study did not report thyroglobulin or calcitonin measurements, limiting insight into potential C-cell activity.

Evidence Regarding Thyroid Malignancy

In contrast to the functional evidence gap, the large-scale cohort studies by Morales et al. [13] and Pasternak et al. [14] provide robust real-world evidence regarding thyroid oncologic safety. Neither study detected elevated risks of thyroid tumors or thyroid cancer compared with active comparators (sodium-glucose cotransporter 2 inhibitors, dipeptidyl peptidase-4 inhibitors, or sulfonylureas). These findings align with external meta-analyses of cardiovascular outcomes trials [18] and pharmacovigilance data.

Absence of TFT Data in RCTs

The three RCTs included in this review [15-17] collectively randomized over 2,900 participants to semaglutide 2.4 mg or placebo, yet none reported measurements of TSH, fT4, T3, or calcitonin. This omission represents a critical missed opportunity, particularly given that the STEP 7 trial included a predominantly East Asian population [15], in whom ethnic differences in thyroid physiology have been documented. Consequently, the generalizability of thyroid safety conclusions to Asian populations remains unknown.

Comparison With External Literature

Contextual evidence from studies not included in this review warrants brief mention. A prospective study by Zhao et al. [19] documented modest TSH reductions without fT4 or T3 changes following liraglutide therapy, suggesting possible central HPT axis effects. Conversely, the LEADER trial analysis by Hegedüs et al. [20] found no clinically meaningful differences in thyroid hormones over 3.8 years. A meta-analysis by Silverii et al. [21] concluded no adverse thyroid function effects across 64 RCTs, though the authors acknowledged substantial heterogeneity in outcome reporting. These external findings neither contradict nor strengthen our primary conclusion, as they were not subject to the same systematic selection criteria.

Mechanistic Considerations 

Several mechanisms have been proposed in the literature to explain potential GLP-1 RA effects on thyroid function [22-24], though none are directly supported by the included studies and all remain speculative. GLP-1 receptors are expressed on human thyroid C-cells (at lower densities than in rodents) and, to a lesser extent, on follicular cells [6]. Activation of these receptors could theoretically modulate thyroid hormone secretion. Additionally, weight loss of the magnitude achieved with semaglutide (12-15% of baseline body weight) is known to influence thyroid hormone economy through adaptive downregulation of the HPT axis, independent of direct GLP-1 receptor agonism [15-17]. Whether the mild fT4 decrements observed by Zhang et al. [12] represent a direct pharmacological effect or an indirect consequence of weight loss cannot be discerned from available evidence.

Clinical Implications

For clinicians prescribing GLP-1 RAs, the available data, limited as they are, do not provide compelling evidence to support routine TFT monitoring in asymptomatic individuals without known thyroid disease. However, this should not be interpreted as confirmation of a true lack of effect; rather, it reflects the current absence of direct evidence, and clinical vigilance remains reasonable until more definitive data emerge. However, the absence of evidence should not be misinterpreted as evidence of absence regarding potential thyroidal effects. Vigilance for symptoms of thyroid dysfunction (fatigue, temperature intolerance, disproportionate weight changes, or neck masses) remains prudent. For patients with personal or family history of medullary thyroid carcinoma or MEN2, the contraindication to GLP-1 RA use persists based on preclinical toxicology studies, despite the absence of confirmed human cases.

Limitations

Methodological and outcome-mismatch bias: Only one of the six included studies directly assessed TFTs as an outcome measure. The remaining five studies evaluated thyroid malignancy or weight loss endpoints without measuring TFTs, creating a fundamental mismatch between the review's primary research question and the outcomes reported in the included literature. This introduces substantial ascertainment bias and precludes quantitative synthesis through meta-analysis. While conducted using validated tools (RoB 2 and ROBINS-I), risk of bias judgments remain inherently subjective and dependent on the completeness of reporting in the primary manuscripts. The exclusion of studies not published in English and the restriction to peer-reviewed literature may have introduced bias, though the comprehensive search strategy across multiple databases and trial registries mitigates this concern. A protocol was not registered in PROSPERO because this review was initiated as an exploratory analysis without a predefined hypothesis regarding the direction or magnitude of effects.

Clinical and statistical limitations: The heterogeneity of study designs, populations, interventions, comparators, and outcome definitions limits the comparability of findings and precludes definitive conclusions regarding the magnitude or direction of any potential effect. The duration of follow-up in the included studies, ranging from 44 weeks to 3.9 years, may be insufficient to detect slowly evolving thyroid pathology or carcinogenic processes with long latency periods. The rapid evolution of the GLP-1 RA landscape, including oral semaglutide and tirzepatide, means the evidence base may not fully reflect the safety profile of newer formulations or dual incretin RAs. The lack of individual participant data precluded subgroup analyses based on age, sex, baseline thyroid function, or concomitant thyroid medication use. Substantial methodological heterogeneity and the absence of TFT data in five of six studies precluded any quantitative synthesis.

## Conclusions

This systematic review identifies a critical evidence gap: only one of six included studies directly assessed thyroid function tests. Mild fT4 reductions were reported in that single study, but causal evidence is lacking, and no RCTs have systematically measured TFTs. For asymptomatic patients without known thyroid disease, routine TFT monitoring during GLP-1 RA therapy is not strongly supported by the currently available evidence, though this conclusion derives from a limited body of direct data and may evolve as more rigorous studies emerge. However, clinicians should remain vigilant for symptoms of thyroid dysfunction (fatigue, temperature intolerance, disproportionate weight changes, or neck masses). The contraindication for patients with personal or family history of medullary thyroid carcinoma or MEN2 remains. Reassuringly, large-scale cohort studies demonstrate no increased risk of thyroid malignancy with these agents. Prospective TFT assessments should be incorporated into ongoing and planned GLP-1 RA trials, accompanied by long-term registries to monitor thyroid outcomes in real-world populations. Until such evidence accrues, clinicians should practice informed vigilance rather than routine laboratory screening.

## References

[1] Alharbi AG (2025). GLP-1 receptor agonism: a transformative approach for managing type-2 diabetes and obesity. Saudi Pharm J.

[2] Alzahrani AM, Alshobragi GA, Alshehri AM (2025). Molecular pharmacology of glucagon-like peptide 1-based therapies in the management of type two diabetes mellitus and obesity. Integr Pharm Res Pract.

[3] Sagliocchi S, Restolfer F, Cossidente A, Dentice M (2024). The key roles of thyroid hormone in mitochondrial regulation, at interface of human health and disease. J Basic Clin Physiol Pharmacol.

[4] Van Uytfanghe K, Ehrenkranz J, Halsall D, Hoff K, Loh TP, Spencer CA, Köhrle J (2023). Thyroid stimulating hormone and thyroid hormones (triiodothyronine and thyroxine): an American Thyroid Association-commissioned review of current clinical and laboratory status. Thyroid.

[5] Kupnicka P, Król M, Żychowska J, Łagowski R, Prajwos E, Surówka A, Chlubek D (2024). GLP-1 receptor agonists: a promising therapy for modern lifestyle diseases with unforeseen challenges. Pharmaceuticals (Basel).

[6] Espinosa De Ycaza AE, Brito JP, McCoy RG, Shao H, Singh Ospina N (2024). Glucagon-like peptide-1 receptor agonists and thyroid cancer: a narrative review. Thyroid.

[7] Kelly CA, Sipos JA (2025). Approach to the patient with thyroid nodules: considering GLP-1 receptor agonists. J Clin Endocrinol Metab.

[8] Page MJ, McKenzie JE, Bossuyt PM (2021). The PRISMA 2020 statement: an updated guideline for reporting systematic reviews. BMJ.

[9] Amir-Behghadami M, Janati A (2020). Population, Intervention, Comparison, Outcomes and Study (PICOS) design as a framework to formulate eligibility criteria in systematic reviews. Emerg Med J.

[10] Sterne JA, Savović J, Page MJ (2019). RoB 2: a revised tool for assessing risk of bias in randomised trials. BMJ.

[11] Sterne JA, Hernán MA, Reeves BC (2016). ROBINS-I: a tool for assessing risk of bias in non-randomised studies of interventions. BMJ.

[12] Zhang Z, Yang J, Gao L (2026). Implications of glucagon-like peptide-1 receptor agonists on thyroid function and thyroid nodules: a drug target Mendelian randomization and cohort study. Endocr Pract.

[13] Morales DR, Bu F, Viernes B (2025). Risk of thyroid tumors with GLP-1 receptor agonists: a retrospective cohort study. Diabetes Care.

[14] Pasternak B, Wintzell V, Hviid A (2024). Glucagon-like peptide 1 receptor agonist use and risk of thyroid cancer: Scandinavian cohort study. BMJ.

[15] Mu Y, Bao X, Eliaschewitz FG (2024). Efficacy and safety of once weekly semaglutide 2.4 mg for weight management in a predominantly east Asian population with overweight or obesity (STEP 7): a double-blind, multicentre, randomised controlled trial. Lancet Diabetes Endocrinol.

[16] Wadden TA, Bailey TS, Billings LK (2021). Effect of subcutaneous semaglutide vs placebo as an adjunct to intensive behavioral therapy on body weight in adults with overweight or obesity: the STEP 3 randomized clinical trial. JAMA.

[17] Wilding JP, Batterham RL, Calanna S (2021). Once-weekly semaglutide in adults with overweight or obesity. N Engl J Med.

[18] Bethel MA, Patel RA, Merrill P (2018). Cardiovascular outcomes with glucagon-like peptide-1 receptor agonists in patients with type 2 diabetes: a meta-analysis. Lancet Diabetes Endocrinol.

[19] Zhao N, Wang X, Wang Y, Yao J, Shi C, Du J, Bai R (2021). The effect of liraglutide on epicardial adipose tissue in type 2 diabetes. J Diabetes Res.

[20] Hegedüs L, Sherman SI, Tuttle RM, von Scholten BJ, Rasmussen S, Karsbøl JD, Daniels GH (2018). No evidence of increase in calcitonin concentrations or development of C-cell malignancy in response to liraglutide for up to 5 years in the LEADER trial. Diabetes Care.

[21] Silverii GA, Monami M, Gallo M (2024). Glucagon-like peptide-1 receptor agonists and risk of thyroid cancer: a systematic review and meta-analysis of randomized controlled trials. Diabetes Obes Metab.

[22] Bjerre Knudsen L, Madsen LW, Andersen S (2010). Glucagon-like peptide-1 receptor agonists activate rodent thyroid C-cells causing calcitonin release and C-cell proliferation. Endocrinology.

[23] Bea S, Son H, Bae JH, Cho SW, Shin JY, Cho YM (2024). Risk of thyroid cancer associated with glucagon-like peptide-1 receptor agonists and dipeptidyl peptidase-4 inhibitors in patients with type 2 diabetes: a population-based cohort study. Diabetes Obes Metab.

[24] McClendon KS, Riche DM, Uwaifo GI (2009). Orlistat: current status in clinical therapeutics. Expert Opin Drug Saf.

